# Gut Mucosal Antibody Responses and Implications for Food Allergy

**DOI:** 10.3389/fimmu.2018.02221

**Published:** 2018-09-27

**Authors:** Ramona A. Hoh, Scott D. Boyd

**Affiliations:** Department of Pathology, Stanford University, Stanford, CA, United States

**Keywords:** gastrointestinal mucosa, B cell, plasma cell, food allergy, plasma cell, antibody repertoire, IgE, memory

## Abstract

The gastrointestinal mucosa is a critical environmental interface where plasma cells and B cells are exposed to orally-ingested antigens such as food allergen proteins. It is unclear how the development of B cells and plasma cells in the gastrointestinal mucosa differs between healthy humans and those with food allergy, and how B cells contribute to, or are affected by, the breakdown of oral tolerance. In particular, the antibody gene repertoires associated with symptomatic allergy have only begun to be characterized in full molecular detail. Here, we review literature concerning B cells and plasma cells in the gastrointestinal system in the context of food allergy, with a focus on human studies.

## Food allergy

The healthy physiological response to dietary antigen exposure in the gastrointestinal tract is a state of immunological non-responsiveness called oral tolerance. The development of IgE-mediated sensitization to allergens, and symptomatic food allergy, can be considered as evidence of a breakdown in oral tolerance, but the mechanisms leading to these pathological outcomes in humans are not fully understood (1–3). In this review, we focus on IgE-mediated food allergies, in contrast to other pathological responses to dietary antigens such as celiac disease responses to gluten. Food allergy affects 3–8% of the US population, and its prevalence is increasing (4). Allergy to peanuts is especially common, affecting up to 1% of the US population and up to 3% of children, and is distinct due to its severity and persistence into adulthood to a greater extent than egg, milk, wheat, and soy allergies (5). Avoidance is the current standard of care (6), and no diagnostic tests can accurately predict an individual's risk for anaphylaxis or the threshold allergen dose. Clinical trials have demonstrated the efficacy of allergen-specific immunotherapy to desensitize against food allergy, as evaluated in a recent meta-analysis of 25 randomized and 6 non-randomized studies, with a total of 1,259 patients treated via oral, sublingual, and epicutaneous routes, and assessment of clinical outcomes of desensitization in 27 studies and sustained unresponsiveness in 8 studies (7). Almost all studies in this meta-analysis showed increased efficacy of immunotherapy compared to controls, albeit with increased risk of adverse events during the trials. Whether the mechanisms leading to desensitization overlap with those of normal oral tolerance is less clear. There have been significant methodological challenges to progress in understanding the mechanisms of normal oral tolerance, the development of food allergy, and therapeutic responses to allergy immunotherapies, as well as extrapolating the results from mouse experiments to humans.

In humans, gastrointestinal (GI) tissues are usually procured during diagnostic testing in the context of suspected GI illness, or during autopsy, and are not routinely collected for patients with food allergy, limiting the availability of both healthy, and allergic patient tissues. Mouse models for allergy, although enabling well-controlled perturbation of immune function, and study of all tissues, have significant limitations. Different models vary according to strain in their propensity to develop IgE responses, and can require non-physiological methods of sensitizing mice to allergens, such as damaging the mouse GI tract with toxins (8, 9). More recently, improved humanized mouse models supporting human immune cell populations have addressed some of these limitations (10).

Despite these challenges, a combination of human and mouse studies has revealed some important themes in the loss of tolerance to food allergens, and the development of symptomatic food allergy. The “barrier regulation” hypothesis (11) posits that allergic sensitization begins with the impairment of the epithelial barrier in the skin, GI tract, and potentially other sites. Reasons for impaired barrier function can include mutation of genes encoding the skin and mucosal epithelium barrier protein filaggrin (12) or the SERPINB (serine protease inhibitor B) gene cluster (13). Individuals with food allergies have increased barrier permeability (14–16). In the context of a damaged epithelial barrier, exposure to food allergens through a non-oral route, or in increased quantities, are hypothesized to contribute to allergic sensitization. Indeed, human epidemiological studies indicate that non-oral contact with food allergens (such as exposure to peanut-containing dust in the home) is correlated with a child's risk of developing food allergy (17, 18). Further, symptomatic food allergy is often observed when a child first ingests the allergenic food, consistent with prior sensitization by non-oral routes (2). Allergic sensitization is thought to require the activity of T cells expressing Th2 cytokines such as IL-4 and IL-13, although the exact nature of the T cell help, if any, required to cause allergen-specific B cells to class switch to IgE *in vivo* in humans have not yet been described. Similarly, the extent to which switching to IgE occurs in various tissues of the body is unclear. Additionally, other cell types such as mast cells, which are commonly found in the tissue, secrete IL-4, IL-13, and other cytokines that may influence B cell development (19). Increased titers of high-affinity allergen-specific IgE antibodies are frequently detected in patients with symptomatic allergy. These antibodies bind FcεRI on tissue-resident mast cells and circulating basophils, where they participate in early/immediate hypersensitivity responses when crosslinked by allergens. Allergen-specific IgE has also been reported to contribute to allergy pathogenesis through facilitated antigen presentation and epitope spreading via uptake of antigen-IgE complexes by the low-affinity IgE receptor, CD23, present on dendritic cells, B cells, and other antigen-presenting cells (APCs) (20–24). IgE can also assist in the transport of antigen from the lumen across the epithelium via CD23 on the surface of epithelial cells, as has been demonstrated in human gut (25), cultured human respiratory epithelial cells (26) and a mouse model for allergy (27).

## Anatomical localization of B cells/plasma cells in the gut

The GI tract is the primary interface with dietary antigens, and is composed of immunologically active tissues. It has been estimated that up to 80% of all plasma cells in humans are in the gut, although lower estimates have also been proposed (28, 29). Most B cells in the GI tract are in the gut-associated lymphoid tissue (GALT), which includes the tonsils, adenoids, Peyer's patches of the small intestine, appendix, and lymphoid follicles of the large intestine and rectum. Plasma cells are found in the submucosa of GI tissues, particularly in the layer of loose connective tissue called the lamina propria, as well as the GALT (30, 31). The GALT is separated from the lumen by epithelial cells, which in addition to forming a protective barrier against the gut microbiota and ingested pathogens, also play an important role in transporting secretory IgA antibodies and secretory IgM into the lumen. Much of the gut epithelium is villous, but regions of the epithelium are associated with lymphoid follicles and are called the follicle-associated epithelium (FAE). Lymphatic circulation through the lamina propria of the intestine passes to the mesenteric lymph nodes and lymphoid follicles within the GALT, where antigen presentation and interaction with T helper cells can induce B cell class-switching and affinity maturation to generate an antibody response.

In human and mouse, the majority of antibody-secreting cells (ASCs; plasmablasts and plasma cells) in the GI tract express IgA, with estimates of 75–80% in the gastric mucosa, duodenum and jejunum, and 90% in the colon (32). IgG-expressing ASCs have been reported to represent 13% of ASCs in the gastric mucosa, and 3–4% in the small intestine and large bowel (32). IgM+ ASCs are also detected: 11, 18, and 6% of total ASCs in the gastric mucosa, small intestine and colon are IgM (32). An important knowledge gap in the context of food allergy is the frequency of the more rare IgE+ ASC or B cells in the human GI tract, as this has not been studied systematically and comprehensively using modern methods in either healthy subjects or allergic individuals.

## Development of gut B cells

What is the anatomical origin of the B cells and plasma cells detected in the gut? Most B-lineage cells in lymph nodes and other secondary lymphoid tissues are thought to be derived from precursors that develop in the bone marrow, where they are exposed to self-antigens, and where autoreactive B cells are deleted from the repertoire (33). Do B cells and plasma cells detected in the GI tract share this origin? B cell development outside of the bone marrow has been demonstrated in the rabbit, chicken, sheep, and mouse (34, 35). Wesemann et al. recently determined that rare RAG2-expressing pre-B-cells exist in the mouse intestinal lamina propria, but are absent from Peyer's patches (35). These pre-B-cell populations are upregulated in response to colonization with gut bacteria compared to germ-free mice, but rapidly decrease in frequency after weaning (35). Deep sequencing of BCR repertoires showed that immunoglobulin heavy chain V_H_ gene segment repertoires were similar between bone marrow and gut, but the immunoglobulin kappa light chain V_K_ gene segment repertoires were distinct, suggesting that repertoire development can be influenced by microbial factors, and that this occurs a critical time window in early life (35). The possibility of B cells originating in human GI tissues early in life is particularly interesting in the context of food allergy, where early feeding with allergenic foods correlates with reduced risk of allergy development (11, 36, 37). Pre-B cells have been detected in the human fetal intestine and other tissues including liver, lung, kidney, and spleen, in addition to bone marrow, as early as 18–20 weeks of gestation (6, 38). Aggregates of B cells have been seen in intestinal tissues by 11 weeks of gestation, but germinal centers in Peyer's patches do not appear until after birth and exposure to the environment (32). Even in adults, transitional B cells can be detected in gut tissue (39), and there is some evidence for lambda light chain revision in mature intestinal B cells (40). More evidence is needed to establish the extent of these developmental pathways in humans, and whether they influence disease states such as food allergy.

## Gut mucosal B cell responses

B cell responses in gut tissues require introduction of antigen from the lumen into the underlying tissue. In healthy mice and humans, oral antigen can be sampled from the lumen by microfold (M) cells that overlay Peyer's patches, by dendritic cells and macrophages, and by intestinal goblet cells and epithelial cells that express antibody receptors that can transport antibody-antigen complexes (41). Antigen transcytosed by M cells and dendritic cells in the FAE is delivered to the Peyer's patches, while antigen that is sampled at the villous epithelium enters the lamina propria, where it is transported through the lymph to the mesenteric lymph nodes (41). Germinal center responses can occur at either of these sites; however, the immune responses may differ qualitatively, as data from mouse indicate that mesenteric lymph nodes are required for oral tolerance, while Peyer's patches are dispensable (42–45). As noted above, prior studies have identified disrupted epithelial cell junctions in allergic patients and those with asthma, suggesting that the routes of antigen exposure in these individuals may differ significantly from healthy subjects (46).

In germinal center reactions B cells can adopt a memory B cell fate or differentiate into plasmablasts that may further differentiate into short- or long-lived plasma cells. Antibody-secreting cells (plasmablasts and plasma cells) are guided to their effector sites in the lamina propria of the GI tract, or through the efferent lymph to the blood and other tissues through expression of homing receptors. The mixtures of homing receptors expressed on the B cells depend on such factors as stimulation from dendritic cells, the site where the ASC was generated, and the isotype of the ASC (47–49). As reviewed in (47), early adoptive transfer experiments in mice found that IgA-expressing ASCs arising from Peyer's patches, mesenteric lymph nodes, and other sites in the GALT traffic to a subset of mucosal sites (intestine, urogenital tract, mammary glands, salivary glands, and respiratory tract), whereas IgA-expressing ASCs from mediastinal and tracheal-bronchial lymph nodes trafficked to the salivary glands and respiratory tract, and rarely to the intestine. In contrast, IgG ASCs preferentially migrate to the bone marrow. Interestingly, Carbon-14 dating experiments indicate that plasma cells can persist for decades in humans, suggesting that, like bone-marrow-resident plasma cells, gut-localized plasma cells could be long-lived, and possibly even provide protective antibody responses for life (50). The combinatorial expression of homing molecules provides one mechanism for the dispersal of locally generated ASCs and B cells to distal humoral and mucosal sites (47), a feature that has appeal for mucosal vaccination strategies (51) and also for food allergy immunotherapy.

## Isotype-switching in the gut

IgE, the causative antibody isotype for human type I hypersensitivity reactions, is generated by isotype switching in B cells expressing constant region genes located upstream of the IgE locus in the genome. Because class switch recombination deletes genomic DNA between the starting isotype and the resultant isotype, the process is unidirectional and irreversible, but it should be noted that within a B cell clone there may be members expressing a variety of different upstream isotypes, including IgM (52, 53). Two major pathways of class-switching to IgE have been described in mouse: direct switching, from IgM to IgE, and sequential switching, from IgM to IgG and then to IgE. Sequential class-switching through an IgG intermediate is clearly not an absolute requirement for IgE generation, as mice carrying deletions of the γ1 intron or γ1 switch regions do not class-switch to IgG1 and are able to generate wild-type levels of IgE (54, 55). However, the extent to which direct class-switching contributes to the generation of IgE under normal conditions is unclear. It has been proposed that high affinity IgE is generated through class-switch from IgG1 memory B cells, which act as the storage for IgE memory (56). However, evidence for memory IgE+ B cells in humans has recently been obtained by Sicherer et al. (4) and Heeringa et al. (57). Employing a negative gating fluorescence-activated cell-sorting strategy, the authors identified a population of IgE+ memory B cells that was higher in frequency in young individuals with food allergy, atopic dermatitis and/or asthma relative to non-allergic controls. IgE+ plasmablasts had fewer somatic hypermutations and less evidence for antigen selection than the IgE+ plasmablasts from healthy controls, potentially indicating that these cells may not give rise to high-affinity IgE+ antibodies. Correlation of allergen-specific binding and affinity of IgE derived from plasmablast populations and putative memory B cell populations, if these can be obtained, may help to clarify the relevance of these populations to disease phenotypes, in future research.

Most of what we know about IgE production in humans derives from studies of primary or cultured cells from secondary lymphoid sites such as lymph nodes, adenoids and tonsils, and rare circulating IgE+ B cells that appear in the peripheral blood (4, 53, 58–63). In humans, data from these tissues suggests that the precursors of IgE are often IgG-expressing B cells, with smaller contributions from IgM-expressing B cells (4, 58, 64). Importantly, there is also some published evidence for local production of IgE class-switched B cells in non-lymphoid tissues. Data from measurements of IgE constant region germline transcripts (εGT), mature IgE transcripts, class-switch recombination (CSR) excised DNA circles and switch circle transcripts, and the expression of factors required for CSR such as IL-4, IL-13 and AID, have indicated that local class-switching to IgE can occur in a variety of non-lymphoid tissues (52, 64–66). IgE class switching has been reported in human bronchial mucosa of patients with atopic and non-atopic allergy (65), in the sinus mucosa of individuals with chronic sinusitis (66), and in nasal polyps of individuals with chronic rhinosinusitis (64). More recently, high-throughput sequencing has been used to identify the nasal mucosa as a tissue site that harbors reservoirs of allergen-specific IgE and clonally-related B cells expressing other non-IgE isotypes in individuals with seasonal allergies (52). Additional perspectives on the evidence for pathways of IgE development in human and mouse, including controversies regarding IgE memory in mice, have recently been published (67–72).

What is known about class-switching in the GI tract in mice and humans? The regulation and localization of class-switching to IgA in the gut, and its importance in maintaining homeostasis with gut commensals, has been studied extensively [reviewed in (73)]. Recent mouse research has demonstrated that class-switching to IgA predominantly occurs in the epithelium-proximal region of the Peyer's patches termed the sub-epithelial dome (74). The authors found that IgA class switching was TGFß-dependent and required interaction of activated B cells with dendritic cells; migration to the subepithelial dome was promoted by CCR6 upregulated in Peyer's patch B cells in response to CD40 signaling. Pre-GC and memory B cells were both found to have high expression levels of CCR6, suggesting “privileged access” of some B cell subtypes to the SED, where microfold (M) cells in the FAE transcytose luminal antigens.

Much less is known about the development of IgE in the gut. Secreted IgE antibodies are detectable in intestinal juices (75, 76) and in the stools (77) of adults with food allergy, suggesting antibody production by local IgE+ plasma cells. Milk-specific IgE has also been detected in the stools of children with milk allergy (78). The numbers, cellular phenotype, and origin of IgE-expressing B cells in the human gut, however, are poorly described. Early studies employing immunofluorescence staining approaches detected IgE+ cells in human stomach, small intestine, colonic, and rectal mucosa, but due to limitations in immunofluorescence staining for other markers to prove cell lineage identity, it was not determined whether these IgE+ cells were B cells, plasma cells, or instead were mast cells or basophils bearing soluble IgE antibodies (79–81). Analysis of intestinal biopsies from 25 patients with food allergy and 14 healthy controls has identified εGT in gut intestinal mucosa (82). This study found that εGT and IL-4 levels were higher in biopsies from food allergic individuals compared to controls, but that εGT could also be detected in a subset of healthy individuals. A recent study of 11 pediatric patients with the allergy-associated condition eosinophilic esophagitis (EOE), and 8 healthy controls, found that total B cells were present at elevated densities in samples from the EOE patients, and that IgE constant region germline transcripts were elevated in EOE esophageal biopsies (83). This study further detected mature IgE mRNA transcript levels in esophageal biopsies by PCR amplification and gel electrophoresis, but did not see statistically significant differences in the proportion of positive biopsies in EOE and control groups (83).

## Intestinal microflora and diversification of the antibody repertoire

Constant exposure to microbial contents of the GI tract is likely to provide much of the antigenic stimulus experienced by B cells in these tissue sites. Several studies in humans have linked dysbiosis of the microbiota with the development of allergy [reviewed in (11)]. How do the components of the microbiota shape the antibody repertoire? Initial reports are beginning to address this large topic. Analysis of human colonic IgA sequences from 6 individuals before antibiotic treatment, after 4 days of treatment, and 46 days after the end of the treatment revealed persistent clones whose frequencies were not altered by antibiotic treatment, suggesting that the IgA repertoire is stable under these conditions of altering the microbiome, at least within the short timeframe of the study (84). Data from a large-scale analysis of recombinant natively-paired antibodies produced from mouse intestinal plasma cells and B cells indicate that most IgA are polyreactive with broad specificity for intestinal microbiota, and although they arose from B cells selected into germinal center reactions in Peyer's patches, this process was not dependent upon microbial colonization or upon food antigens, nor was their affinity dependent on the acquisition of somatic hypermutation (85). The conclusions of this study were that polyreactive anti-microbial IgA antibodies are natural antibodies that arise without instruction by the intestinal microbiome. In contrast, a study of human IgA and IgG antibodies cloned from single plasmablasts sorted from ileum lamina propria found that only 26% of IgA antibodies and 26% of IgG antibodies were polyreactive, defined as binding to at least 2 antigens in a panel of antigens including double-stranded DNA, LPS, and insulin (86).

What evidence is there for GI microbiota affecting IgE production in mice or humans? It has been reported that germ-free mice develop high IgE serum levels and undergo CD4 T-cell-dependent class-switching to IgE in Peyer's patches, suggesting that normal microbiota can help to suppress the levels of IgE-expressing B cells or plasma cells (87). Although not directly addressing the question of allergen-specific IgE production, one study with a mouse model for peanut allergy demonstrated that Clostridia-containing microbiota were associated with class-switching to IgA, which was proposed to have a protective effect against sensitization to allergens. Consistent with this hypothesis, increased rates of peanut allergen protein Ara h 2 and Ara h 6 uptake and appearance in the serum were observed in *Rag–/–* knockout mice, which are unable to generate B cells (88).

## Antibody gene repertoires and clonal relationships in the gut and other tissues

Immunological analyses of humans have typically relied on analysis of the peripheral blood, as these specimens are readily obtained with low risk to research participants, but it is an open question whether the B cells in the blood can provide an adequate sampling of those in other tissue sites. A variety of studies have examined the extent to which B cells clonally related to those in the gut appear in other tissues such as the peripheral blood (89), bone marrow (90), and mammary glands (84); other work has addressed the extent of dispersal of members of B cell clones between different sites in the GI tract (91). A recent study using high-throughput DNA sequencing analyzed B cell clonal distribution between blood, spleen, bone marrow and lung, in comparison to mesenteric lymph nodes, jejunum, ileum, and colon. The GI tissues showed high frequencies of shared clones between tissues, while other clones were found shared at high frequencies between blood, marrow, spleen, and lung, suggesting two major networks of B cell clonal populations (92). Whether this pattern of clonal sharing would hold for B cells and plasma cells expressing different isotypes, such as IgA subtypes compared to IgG subtypes, will require further study, as the data were generated from genomic DNA template from which isotype information cannot be obtained. In investigations of celiac disease, analysis of clonal relationships between plasma cells in distinct intestinal biopsy locations identified significant sharing of clone members between biopsy sites, particularly for the clones expressing antibodies specific for the autoantigen tissue transglutaminase-2 (93). Another analysis of highly abundant IgA-expressing clones in the blood of two human adults found that 36.5% of these selected high-frequency IgA lineages could also be detected in jejunal samples from the same individuals (89). It remains to be seen whether smaller clones are similarly shared between different tissue sites, whether categories of tissue-specific clonal B cell lineages exist in humans, and how anatomical clone sharing relates to the biology of IgE-expressing cell lineages.

BCR repertoire sequencing of flow cytometrically sorted B cell subpopulations has been used to link a population of IgM+ memory B cells with IgM+ plasma cells that produce antibodies that coat commensal bacteria (94). The authors found that, compared to peripheral blood and colon, a high percentage of B cells in the small intestine are memory B cells that express IgM. Memory IgM+ B cells shared clonal overlap with IgM+ plasma cells and, to a lesser extent, IgA+ plasma cells (94). This study also showed that IgM+ memory B cell/plasma cell pools in the gut showed decreased usage of the IgH V genes IGHV1-18, IGHV1-69, and IGHV4-34, and increased usage of IGHV3-7 and IGHV3-23, as has been observed in antigen-experienced, somatically hypermutated peripheral blood B cell repertoires (95). Compared to naïve B cells sorted from the gut, gut IgM memory and plasma cell subsets have more mutated IGHV gene regions and shorter CDR-H3 lengths, providing further evidence for antigen experience (96, 97).

## Summary and outstanding questions

**Figure 1:**
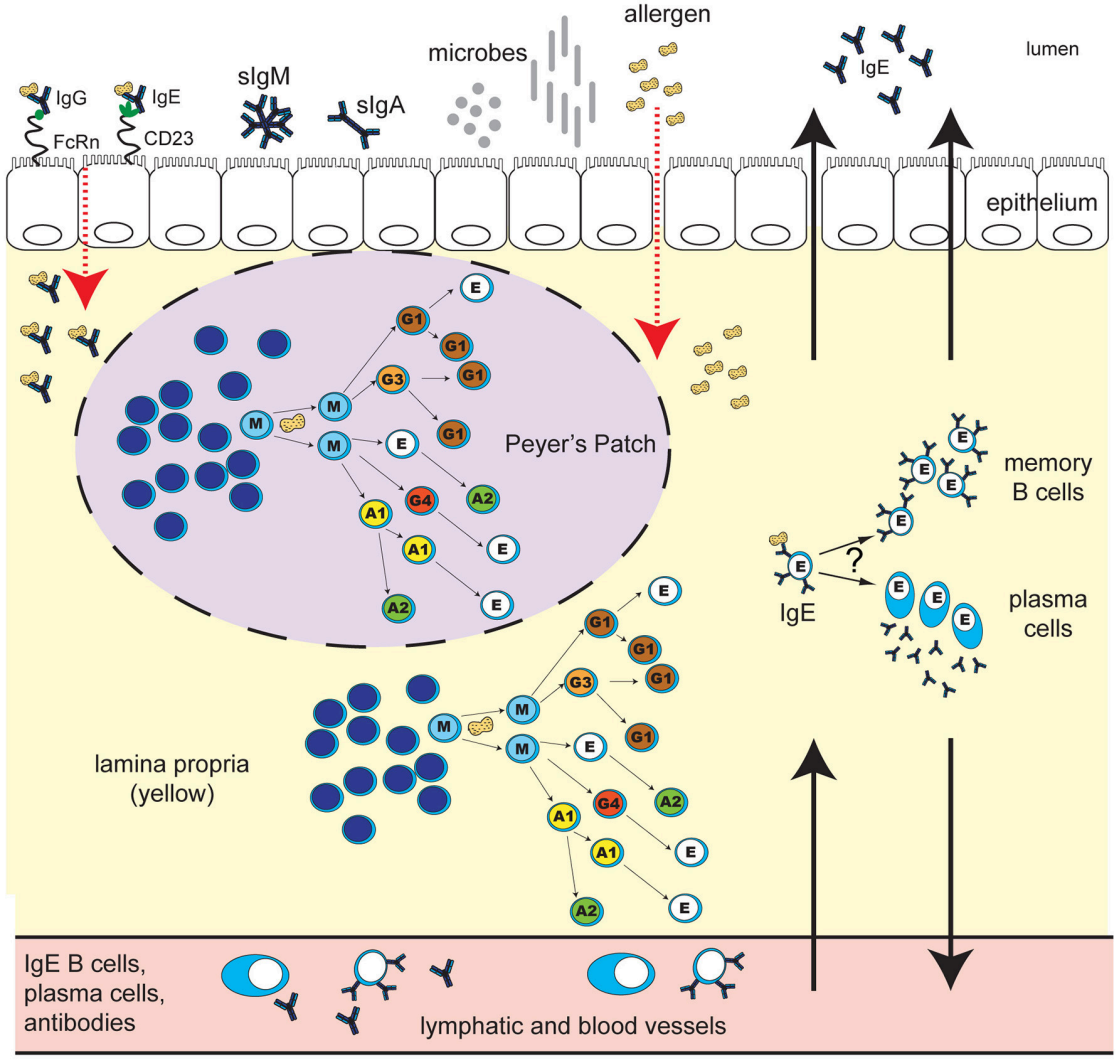
Human IgE development in food allergy: unresolved questions. B cell class-switching to IgE in humans may occur from IgM, IgD, IgG3, IgG1, IgA1, or IgG4, but not from IgA2, which is downstream of IgE in the chromosomal locus. The frequency of class-switching to IgE in the gastrointestinal tract, and the microanatomical localization of these events, is unclear. Potential sites of IgE development include secondary lymphoid structures such as Peyer's Patches (purple oval with dashed line), extrafollicular sites in the lamina propria (yellow), or alternatively, other secondary lymphoid or mucosal sites in the body. What T cell help, if any, is required for IgE switching in different gastrointestinal sites is unclear. B cell exposure to food allergen proteins can occur via introduction of allergen proteins to the GALT, and possibly other tissue sites. The key routes of allergen access from the gut lumen may include epithelial barrier disruption, or transcytosis of immunoglobulin-allergen complexes via Fc receptors expressed on epithelial cells (pictured: allergen-IgE bound to “low-affinity” IgE receptor CD23, and allergen-IgG bound to FcRn). Uptake can also occur via dendritic cells and macrophages (not shown). Key questions for ongoing research include: Do B cells that class-switch to IgE in the gastrointestinal tract differentiate into memory B cells or plasma cells *in situ*? To what extent do daughter cells persist locally in the tissue as opposed to distributing to other sites in the body via the lymph and blood? What are the effector functions of locally-generated vs. systemic food-allergen-specific IgE? How do the microbiota influence these processes? How does the development of IgE in the gastrointestinal tract differ in individuals with food allergy compared to healthy individuals, and how are these pathways altered during immunotherapy for food allergy?

Despite significant progress in increasing our understanding of B cell and plasma cell populations in the tissues of the GI tract, and ongoing enthusiasm for human studies motivated by clinical questions, a number of mysteries about the role of these cell populations in healthy immunity and in food allergy remain unanswered (Figure 1). Detailed analysis of the molecular and cellular mechanisms that guide mucosal IgE-related B cell and plasma cell development and antibody production offers the prospect of answering many of these key questions, particularly in the context of large, well-controlled clinical trials of allergy immunotherapy interventions. Integration of mechanistic studies in clinical trials could aim to address the following areas: determining whether monitoring of B cells/plasma cells in the gut compared to cells in the blood identifies better correlates of successful clinical outcomes or risk of adverse events; understanding how B cell and plasma cell phenotypes in blood, GI tract or bone marrow contribute to serologic changes that occur during immunotherapy, particularly in allergen-specific IgE, IgG4, and IgA; assessing the effect that microbiome modifications may have on gut B cells and plasma cells, and their relationship to therapeutic outcomes. Inclusion of GI biopsies in immunotherapy trials will be required to provide a starting point for addressing these questions. Insights obtained from such studies may lead to new strategies for the prevention, diagnosis, and treatment of IgE-mediated food allergy.

## Author contributions

All authors listed have made a substantial, direct, and intellectual contribution to the work and approved it for publication.

### Conflict of interest statement

The authors declare that the research was conducted in the absence of any commercial or financial relationships that could be construed as a potential conflict of interest. The handling Editor declared a shared affiliation, though no other collaboration, with the authors [SB and RH].
